# Cutaneous Squamous Cell Carcinoma in Immunocompromised Patients

**DOI:** 10.3390/cancers17213476

**Published:** 2025-10-29

**Authors:** Song Hon Hwang, Maie St. John

**Affiliations:** 1 Department of Head & Neck Surgery, David Geffen School of Medicine, University of California Los Angeles, Los Angeles, CA 90095, USA; songhonhwang@mednet.ucla.edu; 2 Department of Otolaryngology—Head & Neck Surgery, Johns Hopkins School of Medicine, Johns Hopkins University, Baltimore, MD 21205, USA

**Keywords:** cutaneous squamous cell carcinoma, immunosuppression, non-melanoma skin cancer, immunotherapy, organ transplantation, clinical trials, cutaneous malignancy

## Abstract

**Simple Summary:**

Cutaneous squamous cell carcinoma is a common skin cancer with increasing prevalence worldwide. While most patients do very well with simple surgical excision, immunosuppressed patients experience worse outcomes. Current staging and treatment guidelines are primarily based on studies from immunocompetent patients, making the management of immunocompromised individuals challenging. Growing understanding of the immune system, tumor microenvironment, and tumorigenesis has shed light on promising new therapeutic modalities. In this review, we summarize the current evidence on the pathogenesis, staging, treatment, and clinical trials of cutaneous squamous cell carcinoma in immunocompromised patients.

**Abstract:**

Cutaneous squamous cell carcinoma (cSCC) is the second most common skin malignancy in the world, representing approximately 20% of all skin cancers. Immunosuppression is a well-established risk factor, contributing not only to the development of new cSCC lesions but also to more aggressive disease and increased mortality. Despite the National Comprehensive Cancer Network (NCCN) and the American Joint Committee on Cancer (AJCC) 8th edition updates recognizing immunosuppression as a risk factor for cSCC, standardized management protocols for these high-risk patients remain limited. As a result, treatment of this already high-risk group remains a significant challenge and highlights the need for dedicated research and attention to improve outcomes in this patient population. This review explores the current knowledge regarding cSCC in IS patients, outlines key gaps in the knowledge, and highlights recent clinical trials to further guide the evaluation and management of these patients.

## 1. Introduction

Nonmelanoma skin cancer (NMSC), primarily composed of basal cell carcinoma (BCC) and cutaneous squamous cell carcinoma (cSCC), is the most common malignancy worldwide [1]. While historically cSCC accounted for approximately 20% of skin cancers, recent studies suggest a dramatic increase in the incidence of cSCC, with one study reporting a 1:1 ratio between BCC and cSCC according to recent Medicare data [2,3,4]. This increase is likely multifactorial, with an aging population and an increase in skin cancer screening being major contributors [5]. The rise in cSCC poses a significant public health concern and highlights the importance of developing effective strategies to address this growing burden.

Classic risk factors for cSCC development are ultraviolet (UV) radiation, age, skin type, male gender, and immunocompromise. The most common site of cSCC is the head and neck, which makes management especially challenging due to functional and cosmetic concerns in this region [6]. Although the vast majority of head and neck cSCC can be treated with simple excision, there is a significant group of patients with more aggressive disease who require additional management [7]. One such subset of patients is those who are immunosuppressed (IS). These patients have an increased risk of developing cSCC as well as potentially worse outcomes compared to the general population [8,9,10,11,12,13]. In organ transplant recipients (OTRs) on chronic immunosuppressive medication, there is a 65- to 250-fold increased risk of developing cSCC, whereas patients with chronic lymphoid malignancies have an 8-fold increase [14,15,16]. For OTRs the risk of cSCC increases by 5% annually after transplantation, and within 20 years, it is estimated that 75% of patients will be diagnosed with cSCC [17,18,19]. Understanding the development and progression of cSCC in IS patients is important, as these patients have been reported to experience differences in prognosis and response to treatment. Locoregional recurrence (LR), nodal involvement, distant metastasis, disease-specific survival (DSS), and overall survival (OS) have all been shown to be significantly worse in IS patients with cSCC [12,20,21].

Although the importance of treating IS patients with cSCC is widely acknowledged, the exact role of immunosuppression on disease pathogenesis and outcomes remains unclear. Studies in this patient population are limited by significant patient heterogeneity and small patient cohorts. This limitation is further compounded by the absence of cSCC tracking in the CDC’s National Cancer Registry, complicating accurate epidemiological data and research. Consequently, the two most widely used staging systems for cSCC, the American Joint Committee on Cancer (AJCC) 8th Edition and the Brigham and Women’s Hospital (BWH) staging system, do not incorporate immunosuppression as a factor in their staging criteria [22,23]. This makes the management of cSCC in IS patients particularly challenging.

The overarching goal of this review is to provide the head and neck physician with an evidence-based framework for managing cSCC in IS patients. We aim to do this by reviewing the current literature regarding immunosuppression, pathophysiology, staging, and overall management of this group of patients and highlighting recent and ongoing clinical trials.

## 2. Defining Immunosuppression

Immunosuppression is a state of impaired immune function that can be acquired or inherited. This leads to a reduction in an individual’s ability to mount an effective immune response, particularly to infections and malignancies. A healthy immune system is able to recognize and eliminate cancer cells through a process called immunosurveillance [24]. IS patients have dysregulated immunosurveillance and, consequently, are more susceptible to various malignancies, most notably cSCC.

According to the National Health Interview Survey, the prevalence of immunosuppression among US adults more than doubled from 2013 to 2021, increasing from 2.7% to 6.6% [25]. This trend may reflect the growing population of IS patients with organ transplants as well as increased screening of immunosuppressive conditions in light of the COVID-19 pandemic [25]. According to data from the Organ Procurement and Transplantation Network, the number of solid organ transplants has increased annually, and in 2024 there was a record-breaking 48,149 organ transplants in the US. The majority of these patients will be placed on chronic immunosuppressive medications that will increase their risk of cSCC by 65- to 250-fold [16,26]. These changes underscore the need for precise identification and tailored treatment strategies in this underrepresented population.

Among IS patients, OTRs are the most extensively studied. However, the risk of cSCC varies greatly within OTRs. As the risk of cSCC is directly proportional to the amount of immunosuppressive therapy, thoracic organ transplantation carries a significantly higher risk of cSCC than renal transplantation [17,27]. Risk may further be increased in lung transplant recipients with the use of voriconazole, a commonly used antifungal prophylaxis, which has been associated with photosensitivity and a 73% elevated risk of developing cSCC [28]. Amongst OTRs, race also influences cSCC development. A retrospective analysis by Pritchett et al. found that the risk factors for cSCC differ in Black and Asian patients, with human papillomavirus (HPV) contributing more in Black individuals. Additionally, the study found that lesions in Black patients were more likely to be in sun-protected areas vs. sun-exposed areas, as seen in Asian and White patients. The authors conclude that nonwhite OTRs remain at risk for cSCC, although their etiology and presentation may differ [14].

Hematopoietic malignancies represent another subset of IS patients. Patients with chronic lymphocytic leukemia (CLL) have a well-documented association with cSCC, and both the disease itself and its treatments can cause significant immunosuppression [29,30,31]. Consequently, CLL patients are at greater risk of developing cSCC. In these patients, cSCC has been shown to have more aggressive behavior and lead to poorer outcomes than in the general population [30]. Multiple myeloma (MM) is a plasma cell malignancy that has been associated with immunosuppression. With the advent of novel therapies, overall survival of MM patients has markedly improved [32]. However, longer survival has been accompanied by higher rates of secondary primary malignancies, most notably cSCC, for which patients with MM demonstrate a 2.44 higher risk compared to the general population [33,34].

Additional sources of immunosuppression include autoimmune diseases, chronic kidney disease, human immunodeficiency virus (HIV), and diabetes mellitus (DM). Although each has been associated with cSCC, the supporting evidence is limited. Patients with rheumatoid arthritis (RA) and psoriasis have both been linked with an increase in NMSCs; however, this effect is shown in the context of tumor necrosis factor (TNF) alpha inhibitor use, which is normally reserved for moderate to severe disease. A large observational study in the US found that the use of biologics in RA patients was associated with an overall risk of 1.5 for NMSCs, while another retrospective analysis found the use of TNF-alpha inhibitors increased NMSC rates by 42% in patients with psoriasis [35,36].

HIV is a well-known cause of immunosuppression and is strongly associated with Kaposi sarcoma. Its association with other cutaneous malignancies is less clear, although recent studies indicate an increased risk of both Merkel cell carcinoma (13-fold) and cSCC (2–5-fold) [37,38,39,40,41,42]. The evaluation of HIV patients is complex and should consider the patients’ antiretroviral therapy, CD4 counts, and viral load. Lastly, type II diabetes mellitus is an immunosuppressive state that has been studied in the context of cSCC development. Although several retrospective studies found an increased risk of NMSCs in long-term diabetic patients, more recent data contradict this claim, and further studies are needed to explore this association [43,44,45].

Current studies investigating IS patients with the cSCC group all immunosuppressed patients into a single cohort [12,46,47,48,49,50,51,52,53]. In a systematic review and meta-analysis, Elghouche et al. concluded that immunosuppression was associated with a worse prognosis in regard to LR, disease-free survival, DSS, and overall survival. Likewise, a large seven-year prospective study by Rosenthal et al. found immunosuppression to be an independent predictor of poor outcomes—defined by tumor recurrence, nodal involvement, regional and distant metastasis, and DSS—in patients with cSCC treated with Mohs micrographic surgery (MMS) [12]. These findings are convincing and show a strong relationship between immunosuppressed status and adverse outcomes. However, as highlighted in this section, IS patients represent a highly heterogenous cohort, and pooled analyses may obscure notable differences between patients. As such, individual patients should be carefully evaluated in the context of their specific cause of immunosuppression to ensure the most appropriate care.

## 3. Pathogenesis of cSCC

cSCC arises from keratinocytes through an accumulation of genetic and epigenetic alterations. UVB-induced DNA damage is the most common cause and often leads to the formation of pyrimidine dimers from C to T and CC to TT transition mutations [54,55]. These mutations most commonly occur in the TP53 gene, a well-known tumor suppressor, and lead to inactivation of the p53 protein. Notably, there is a high prevalence of p53 mutations in normal sun-exposed skin (74%) as well as in actinic keratoses (AKs) and cSCC in situ (40%), suggesting loss of p53 early in the tumorigenesis process [54,56,57]. The estimated annual rate of malignant transformation from AKs to cSCC is 0.025–16% per lesion, and the average patient has 6–8 lesions [58,59]. Therefore, patients with multiple premalignant AKs should be monitored carefully.

Other notable alterations include loss-of-function mutations in the NOTCH pathway leading to dysregulation of epidermal differentiation. Specifically, NOTCH1, a downstream effector of p53, has been shown to be frequently mutated in cSCC and sun-exposed skin [60,61]. Cyclin-dependent kinase inhibitor 2A (CDKN2A), which encodes the p16 tumor suppressor protein, and the RAS family of proteins (HRAS, NRAS, KRAS) are also frequently mutated in AKs and cSCCs [60,62,63]. cSCC has one of the highest mutational burdens amongst malignancies, carrying an approximately 4-fold greater mutational load than melanoma [64]. While an exhaustive review of the genetic alterations is beyond the scope of this review, a working understanding of the molecular drivers is important in this era of targeted therapy.

Currently, there are no targeted therapies for cSCC; however, several agents are under investigation, including epidermal growth receptor factor (EGFR) inhibitors and programmed cell death protein 1 (PD-1) inhibitors [65,66,67,68,69]. Additionally, gene expression profiling has been demonstrated to improve risk stratification and treatment selection in melanoma patients and has prompted a similar 40-gene expression profile assay for cSCC to be developed [70,71]. These novel and promising tools underscore the need for a solid foundational understanding of cSCC pathogenesis.

## 4. Role of the Immune System in cSCC Pathogenesis

The interplay between the immune system and cSCC is complex. This section provides a concise overview to help clinicians assess IS patients and understand the implications of the immune system and their underlying disease.

Both the innate and adaptive immune systems help prevent malignant transformation in the skin by recognizing and eliminating transformed keratinocytes through a process termed immunosurveillance. During cSCC development, malignant cells adapt and undergo immune escape through a process termed immunoediting [24,72].

Immunoediting is further broken down into three phases: elimination, equilibrium, and escape. The elimination phase is protective and involves both natural killer cells and CD4+ and CD8+ T lymphocytes working together to detect and destroy cancer cells. The equilibrium phase is characterized by residual tumor cells that persist but remain constrained by the immune system. During this phase, clearance of malignant cells can lead to positive selection for cells that are resistant to immune clearance via loss of tumor-specific antigens [73]. The final escape phase is characterized by immune system exhaustion either through tumor cell adaptation or immunosuppression and ultimately leads to tumor progression [24,72].

Through the process of immunoediting, tumors foster a tumor microenvironment (TME) that is favorable for tumor growth and immune evasion. Cytokine networks, primarily TGF-beta, IFN-gamma, and TNF-alpha, reprogram innate immune cells into tumor-promoting phenotypes [74,75,76,77]. Key mediators of this process include myeloid-derived suppressor cells, tumor-associated macrophages, and natural killer cells [78]. This process is further complicated by the development of chronic inflammation, which is commonly seen with keratinocyte injury and cSCC pathogenesis. Inflammatory mediators act in autocrine and paracrine fashion to influence the TME, with stage-specific effects on immune cells during tumorigenesis [73].

T-lymphocytes play a key role in antitumor immunity, as they are a major part of the TME immune infiltrate [79]. T-cells are activated by antigen-presenting cells and undergo subsequent cytokine-driven differentiation. The Th1 axis, rich in IL-12 and IFN-gamma signaling, promotes CD8+ cytotoxic cells that have the ability to clear malignant cells [80,81,82]. Dysregulation of this pathway can lead to immune exhaustion and suppression, ultimately leading to tumor persistence and progression. The TME is composed of malignant cells and their immunosuppressive cytokines, such as IL-10 and TGF-beta, that mediate T-cell dysfunction. Additionally, dysfunctional CD8+ T-cells show an elevated expression of inhibitory receptors, including programmed death-1 (PD-1), cytotoxic T-lymphocyte-associated antigen-4 (CTLA-4), TIM-3, and LAG-3 [79,83]. The associated ligands for these receptors, particularly programmed death-ligand 1 (PD-L1), are seen in cSCC patients and have been associated with increased risk of metastasis and worse outcomes [84].

Regulatory T-cells (Tregs) are commonly found within the TME in numerous malignancies, including cSCC. Tregs secrete IL-10 and TFG-beta to promote an overall immunosuppressive environment by inhibiting effector responses through both direct and indirect mechanisms [85,86]. Elevated levels of Treg have been shown to suppress Th1-mediated antitumor immunity and are associated with worse prognosis in cSCC [87].

## 5. Clinical Implications of Immunosuppressive Drugs in cSCC

Immunosuppressive drugs represent a diverse class of medications that modulate immune function through distinct mechanisms. These agents are essential in organ transplantation, enabling graft retention through immune suppression. The most common drugs used for organ transplantation are antimetabolites (e.g., azathioprine, mycophenolate), calcineurin inhibitors (e.g., cyclosporine, tacrolimus), corticosteroids (e.g., prednisone), and mammalian target of rapamycin (mTOR) inhibitors (e.g., sirolimus, everolimus).

Studies suggest that the intensity and duration of immunosuppressive therapy are directly correlated with cSCC incidence and mortality [88,89]. In a randomized, prospective study by Dantal et al., renal transplant patients were assigned to either a low-dose cyclosporin regimen or a normal-dose regimen. At 66 months of follow-up, there was no difference in graft function or survival; however, there was a significant increase in all cancers, including skin cancers, in the normal-dose group [90]. Several retrospective studies have confirmed this positive correlation in different organ transplants [91,92,93]. Furthermore, multimodality therapy has been shown to carry a higher risk of NMSCs than high doses of a single drug. In a prospective study, 110 renal transplant patients were randomized into three groups: triple therapy (prednisolone, cyclosporin, and azathioprine), dual therapy with prednisolone and cyclosporin, and dual therapy with prednisolone and azathioprine. Patients receiving triple therapy had a significantly higher incidence of cancer, particularly NMSCs [94]. These findings may help explain the higher risk of cSCC in thoracic organ recipients, who typically require more intensive immunosuppression.

Azathioprine is a purine analog anti-metabolite that inhibits DNA synthesis and is among the earliest drugs used in OTRs [95]. It inhibits proliferation of all leukocytes and acts synergistically with UV-A to increase photosensitization, causing oxidative DNA damage [96,97]. Azathioprine use has been widely associated with an increased risk of cSCC, and OTRs on immunosuppressive regimens including azathioprine are twice as likely to develop cSCC compared to OTRs not taking azathioprine [98,99].

Mycophenolic mofetil and mycophenolic acid are selective inhibitors of purine synthesis that were developed to replace azathioprine. The evidence on overall cancer risk is mixed, but several studies report reduced rates of cSCC with these medications compared with azathioprine [98,100,101]. Due to these findings, many centers have shifted away from azathioprine use in favor of a mycophenolate-based regimen, especially in patients at high risk of developing secondary malignancies.

Cyclosporin and tacrolimus are calcineurin inhibitors (CNIs) that block the effect of calcineurin, resulting in decreased IL-2 production, T-cell activation, and cytotoxic function [102]. Secondarily, CNIs enhance expression of TGF-beta, which has a dampening effect on CD8+ cytotoxic and natural killer cells, supporting an immunosuppressive TME. TGF-beta has also been associated with increased invasiveness and metastatic potential of cancer cells [102,103,104]. Similarly to azathioprine, CNIs have been shown to act synergistically with UV to cause DNA damage [105,106]. Despite these findings, the carcinogenic potential of CNIs remains uncertain, with conflicting findings across studies [98,107]. Overall, CNIs are associated with increased cSCC risk, though the magnitude of this risk differs between tacrolimus and cyclosporine.

mTOR is a serine/threonine kinase that plays a central role in cellular differentiation and growth. mTOR inhibitors, sirolimus and everolimus, represent a newer class of medications that appear to carry a lower carcinogenic risk than CNIs and azathioprine [108,109,110,111]. In a systematic review and meta-analysis by Knoll et al., sirolimus use was associated with a reduced risk of overall malignancy, including NMSCs, in renal transplant patients [112]. One proposed mechanism for this risk reduction is through suppression of vascular endothelial growth factor leading to inhibition of tumor angiogenesis [113].

Steroids were the first drug used for kidney transplants and remain a central immunosuppressive therapy in transplant patients, particularly as a low-dose maintenance agent. Although steroids are widely accepted as being immunosuppressive, there is no strong evidence that steroids alone increase the risk of cSCC [24,114,115].

Knowledge of the commonly used immunosuppressants and their relative carcinogenic profile provides valuable insight into an individual’s risk of cSCC development and should be considered when treating this high-risk patient population.

## 6. Staging of cSCC

The AJCC 8th Edition and the BWH classification systems are the most used staging systems for cSCC in the United States (Table 1). These systems help physicians identify patients at higher risk for recurrence, nodal metastasis (NM), and disease-specific death (DSD). Several studies have compared the prognostic value of the two systems, with the consensus favoring the BWH system.

In a meta-analysis, Schmitt et al. assessed cSCC patients who underwent sentinel lymph node biopsy (SLNB) and found the BWH system to be more accurate at predicting SLNB positivity, with the majority of SLNB-positive cases categorized as T2 according to both systems [116]. In a single-institution cohort study, Ruiz et al. analyzed 459 total patients with HNcSCC and compared the performance of the two systems in predicting poor outcomes—defined by local recurrence (LR), NM, DSD, and overall survival (OS). The study found no difference in LR and OS but found the BWH had higher specificity and positive predictive value for identifying risk of metastasis and DSD [22]. More recently the same group compared the BWH system to the AJCC and the International Union Against Cancer and found the BWH system offered greater distinctiveness, homogeneity, and monotonicity [117].

Use of the AJCC 8th Edition and BWH systems in IS patients should be performed cautiously, as neither system incorporates immunosuppression in their staging, and evidence validating their use in IS patients is limited. In a retrospective cohort study of 58 IS patients (defined as leukemia, lymphoma, monoclonal gammopathy, organ transplantation, or HIV/AIDS), Blechman et al. reported risks of poor outcomes by BWH stage of 1.8% (T1), 9.9% (T2a), 33.3% (T2b), and 100% (T3), and by AJCC 8th Edition of 1.7% (T1), 8.8% (T2), and 36.4% (T3). They concluded that both systems showed comparable distinctiveness, homogeneity, and monotonicity in IS patients as in the immunocompetent cohort [118]. A separate retrospective comparison of AJCC 7th Edition and BWH found that most adverse events occurred in low T-stage tumors and that BWH was better at predicting NM and LR in IS patients [8]. Both studies are limited by their small sample sizes, retrospective single-institutional design, low event rates, and substantial heterogeneity within the IS population.

The generalizability of the current staging systems is limited in IS patients. In a multi-institutional study, Manyam et al. compared the outcomes of IS and immunocompetent patients treated with surgery and radiation therapy for HNcSCC. They found that IS patients experienced higher LR and worse progression-free survival (PFS). On multivariate analysis, immune status was strongly associated with LR and exerted a larger effect than PNI, leading the authors to recommend incorporating immune status into future prognostic models [11,26]. In an eight-year prospective study of 929 cSCC patients undergoing MMS, Rosenthal et al. showed that immunosuppression independently predicted poor outcomes—defined as tumor recurrence, nodal involvement, regional and distant metastasis, and disease-specific mortality—and outperformed PNI, deep invasion, and clinical size ≥ 2 cm in poor outcome prediction. Further subgroup analyses indicated that only transplant patients and patients with hematologic malignancies showed an association with poor outcomes [12]. Consistently, Tam et al. reported higher DSD among patients with hematopoietic malignancies and transplant recipients compared to diabetic patients [119]. This study indicates that risk for poor outcomes varies across IS subgroups, and stratified analysis is important for future studies.

These findings identify immunosuppression as a strong, independent predictor of poor outcomes in cSCC. IS status may carry greater prognostic value than traditional AJCC and BWH high-risk features—such as PNI and deep invasion. Accordingly, traditional staging systems may underestimate risk in IS patients and should be applied with care.

## 7. Prevention and Prophylaxis

Management of cSCC in IS patients starts with prevention. UV exposure is a well-known risk factor for the development of cSCC, and approximately 90% of cSCC presents in sun-exposed areas, such as the head and neck [27]. Consistent application of broad-spectrum, high-SPF sunscreen and sun-protective clothing should be encouraged, as these methods are easily implemented and highly effective. Patients should be discouraged from unnecessary UV exposure, such as artificial tanning, and should be followed by a dermatologist for frequent skin exams to facilitate early detection and treatment [120].

Despite the importance of limiting sun exposure, patient education and knowledge on the importance of prevention are severely lacking. Numerous studies revealed that IS patients showed lower compliance, understanding, and use of sun-protective behaviors than the general population [121,122,123,124,125]. These findings highlight the importance of formal education as well as the need for multidisciplinary cooperation in providing sun protection education for these high-risk patients.

Another preventative strategy is prophylactic treatment of premalignant lesions. In OTRs, several agents have shown promise in treating precancerous lesions. Acitretin is a systemic retinoid that has been shown to reduce the incidence of cSCC and keratotic lesions [126]. A randomized, double-blind, placebo-controlled study showed that Acitretin 30 mg daily for 6 months significantly reduced the occurrence of cSCC and keratotic skin lesions in renal transplant recipients [127]. Although multiple studies confirm the benefit of Acitretin, cessation of the drug has been linked to severe rebound cSCC and relapse of lesions [127,128,129]. Given the need for continued therapy and its significant adverse effect profile, systemic retinoid prophylaxis for cSCC should be employed with caution and only after careful patient selection.

Capecitabine, an oral 5-fluorouracil (5-FU) prodrug, and topical 5-FU can also be considered for cSCC prevention. 5-FU is a pyrimidine analog that blocks RNA/DNA synthesis and is used in the treatment of numerous malignancies. Patients given low-dose oral capecitabine were shown to have reduced incidence of NMSCs and AKs in solid OTRs in several small studies [130,131]. In a feasibility study, Hasan et al. demonstrated the use of 5% 5-FU was superior to sunscreen use and 5% imiquimod in the clearance of AKs and prevention of new AKs [132]. These preliminary studies indicate 5-FU-based agents may be useful for cSCC prophylaxis in IS patients and offer a superior safety profile than systemic retinoids. Larger, prospective studies are needed to further elucidate the efficacy and safety of these drugs.

Photodynamic therapy (PDT) is another common method for treating AKs. PDT works by applying a photosensitizer to a field of interest (e.g., premalignant lesions) and subsequently treating the area with a specific wavelength of light. This leads to the production of reactive oxygen species that induce an anti-tumor response and cell death. Evidence of PDT in IS patients is sparse; however, a recent systematic review reported the use of methyl aminolaevulinate-PDT in solid OTRs yielded a 76.4% complete clearance and 90% reduction rate of precancerous lesions. It was also more effective at treating AKs than topical 5-FU and 5% imiquimod [133].

Lastly, when considering prevention of cSCC in OTRs, the immunosuppressive regimen of the patient must be strongly considered. As discussed previously, different immunosuppressive medications come with varying risks of cSCC development. Overall, mTOR inhibitors represent a favorable option, as they have been shown to have good immunosuppression with reduced risk of cSCC in comparison to CNIs [134]. However, no official guideline exists regarding the modification of immunosuppressive regimens in high-risk OTRs. In 2006, an expert consensus panel from Europe recommended moderate reduction in immunosuppression in patients with >25 skin cancers per year or for those with a 10% 3-year risk of mortality from their skin cancer [135]. More recently, an expert consensus of dermatologists from the US and Europe recommended discussion with transplant physicians to reduce or modify immunosuppression in patients who develop ≥10 cSCCs per year or with ≥20% risk of nodal metastasis [136]. These recommendations stress the need for coordinated multidisciplinary care.

## 8. Treatment of cSCC in Immunosuppressed Patients

Surgical resection is the standard of care for management of cSCC. According to the National Comprehensive Cancer Network (NCCN) guidelines, surgical excision with 4- to 6 mm margins is recommended for low-risk cSCC [137]. NCCN guidelines classify all lesions of the head and neck as well as lesions in IS patients as high-risk and recommend either MMS or other forms of peripheral and deep end face margin assessment (PDEMA) resection followed by linear repair, skin grafting, or healing by secondary intention. If complex reconstruction with tissue flaps is required, closure should be delayed until final histologic margins are confirmed negative [137]. However, for IS patients, NCCN guidelines are primarily based on consensus opinion and limited evidence, and management should prioritize a personalized and multidisciplinary approach over strict adherence to guidelines.

Patients with lymphoproliferative disorders, such as CLL, highlight the need for individualized treatment. In a retrospective study for patients undergoing MMS for NMSCs, Mehrany et al. noted dense lymphocytic infiltrates and increased subclinical tumor extension to the margins that obscured accurate histological margin assessment [138]. In a larger review of 2998 NMSC cases, Song et al. found that patients with hematologic malignancy and organ transplants were associated with higher odds of aggressive subclinical extension (ASE) [139]. These findings suggest that, despite NCCN guidelines recommending MMS or PDEMA resection for high-risk cSCC, ASE seen in select IS patients may limit reliable margin assessment.

Therapeutic neck dissection is required for positive lymph node involvement in all patients. Guidelines for clinically and radiographically negative necks are less definitive. Low-risk patients can often undergo surveillance. However, patients with multiple high-risk factors, such as IS and head and neck patients, should be strongly considered for SLNB or elective neck dissection given evidence suggesting their increased rates of LR [17]. SLNB has been shown to detect subclinical disease and improve survival in immunocompetent patients, but the same has not been shown in IS patients. A recent, multicenter retrospective cohort study reported that while SLNB reduced nodal recurrence and improved DSS and OS in immunocompetent patients, these benefits were not observed in IS patients [140].

Radiation therapy is seldom used as a first-line treatment for cSCC, as it is associated with worse disease control and a higher LR rate when compared to surgery [141,142]. However, it may be considered as primary treatment in patients who are not surgical candidates or those who decline surgery. The evidence supporting the use of adjuvant radiation therapy is mostly limited to retrospective studies, and its use in IS patients is unclear. According to a 2009 systemic review, although adjuvant radiotherapy is recommended for high-risk cSCC, the authors found no difference in outcomes between surgical monotherapy and surgery plus radiation therapy in patients with PNI [143]. Still, the current NCCN and American Academy of Dermatology guidelines recommend adjuvant radiation in patients with high-risk features, including PNI, positive margins, large tumors, and evidence of metastatic disease [66,141].

## 9. Systemic Therapy and Future Directions

Systemic therapy is reserved for patients with locally advanced or metastatic cSCC not amenable to surgical resection [144]. Historically, cisplatin-based regimens were used in combination with other chemotherapies (e.g., bleomycin, doxorubicin, 5-FU) for advanced cSCC with varying response rates from 17 to 84% [145,146,147,148,149,150,151]. However, many of these studies are limited by small sample sizes and significant heterogeneity between treatment regimens. Additionally, these regimens failed to show durable response after treatment cessation and demonstrate short OS and PFS [152,153]. Due to the lack of high-quality evidence and significant toxicity profile, cytotoxic chemotherapy is not FDA-approved for the treatment of advanced cSCC and is not considered in the treatment of IS patients [144].

Targeted therapy has been an area of active research for unresectable cSCC. Specifically, epidermal growth factor receptor (EGFR) inhibitors have been investigated for their central role in cell differentiation and proliferation [154]. Up to 80% of cSCCs demonstrate overexpression of EGFR, with even higher rates seen in metastatic cSCC [155,156]. The best-studied EGFR inhibitors are cetuximab, panitumumab, gefitinib, and erlotinib [156,157,158,159,160,161,162]. Although they have shown promising results in immunocompetent patients, they have not been studied in IS patients.

A phase II study investigating cetuximab monotherapy in patients with unresectable cSCC showed a 69% disease control rate with mean OS and PFS of 8.1 and 4.1 months, respectively [156]. Findings from a retrospective study by Montaudie et al. demonstrated similar results, showing a disease control rate of 70% at 12 weeks with OS and PFS of 17.5 and 9.7 months in patients with advanced cSCC [163]. The longer OS and PFS is likely attributed to a significantly higher portion of patients having local disease compared to the phase II trial. Erlotinib and gefitinib inhibit EGFR by blocking tyrosine kinase activity. In phase II studies, both agents failed to show a significant response rate as monotherapy in unresectable cSCC, with gefitinib and erlotinib showing an overall response rate of 16% and 10%, respectively [158,160]. However, when combined with other treatments, such as surgery and adjuvant radiation, both agents showed improvement in their responses, suggesting their use in combination with other treatment modalities [157,164]. EGFR inhibitors generally have a favorable side effect profile, but caution must be used, as they have not been thoroughly studied in IS patients.

As our understanding of the role of the immune system in tumorigenesis has grown, immunotherapy has emerged as a potential therapy for cSCC. PD-1 is an inhibitory receptor present on immune cells that acts as a brake on the immune system. Its associated ligand, PD-L1, is commonly expressed on tumor and antigen-presenting cells. The binding of PD-1 with PD-L1 dampens immune signaling and leads to immune exhaustion and evasion by cancer cells and has been well studied in cSCC [165,166]. A pivotal phase I and II study investigated cemiplimab, a human IgG4 monoclonal antibody for PD-1, in advanced cSCC patients and found overall response rates of 47–50% and meaningful durable response rates of 61–65% [67]. Because of these promising results, the FDA approved the use of cemiplimab in 2018 for patients with metastatic or locally advanced cSCC who are not candidates for curative surgery or radiation. Overall, cemiplimab was well tolerated with a 7% discontinuation rate due to adverse events [67]. However, this clinical trial only included immunocompetent patients, and the safety of immune checkpoint inhibitors (ICIs) has not been established in IS patients. In a recent case series and systematic review, Kumar et al. reported OTRs treated with ICIs had an allograft-rejection rate of 41% with PD-1 inhibitors, and although effective, ICIs carry a substantial risk of graft rejection [167].

Pembrolizumab, another anti-PD-1 monoclonal antibody, was approved for use in recurrent, metastatic, and locally advanced cSCC not treatable with surgery or radiation, based on the KEYNOTE-629 trial (NCT03284424). In this study, patients with locally advanced cSCC and those with recurrent/metastatic cSCC had an overall response rate of 50% and 35%, respectively, with durable responses lasting > 12 months [68,69]. More recently, the FDA approved the use of cosibelimab, an anti-PD-L1 monoclonal antibody, for metastatic or locally advanced cSCC in patients not amenable to surgery. Approval was based on a multicenter trial that investigated 109 patients with metastatic (n = 78) or locally advanced (n = 31) cSCC. The overall response rate was 47% and 48% for metastatic and locally advanced cSCC, respectively. Median duration of response was not reached in the metastatic group and was 17.7 months in the locally advanced group [168,169]. Table 2 summarizes the current immunotherapeutic agents approved for the treatment of cSCC.

Several other ICIs have been studied for use in cSCC, including nivolumab (anti-PD-1), ipilimumab (anti-CTLA04), avelumab (anti-PD-L1), and relatimab (LAG-3). Outside of cemiplimab, pembrolizumab, and cosibelimab, these medications have not been FDA approved, and their use for cSCC is off-label. Although immunotherapy has changed the treatment landscape for many malignancies, the generalizability of its efficacy in IS patients is severely limited. A recently concluded clinical trial found that nivolumab showed poor tumor control and led to significant allograft loss in eight kidney transplant patients with advanced skin cancers on tacrolimus and prednisone immunosuppression [170]. Conversely, a phase I study of cemiplimab in 12 renal transplant patients reported a 46% durable antitumor response rate with no reported graft rejection and concluded that mTOR inhibitors and steroids represent a favorable immunosuppressive regimen for these patients [171]. The conflicting evidence of these two studies highlights the need for larger and more robust randomized control trials to investigate the use of ICIs in IS patients.

Intralesional immunotherapy is an emerging treatment that may provide an alternative for patients who are not surgical candidates or cannot tolerate systemic therapy. A phase I trial (NCT03889912) investigating low-dose intralesional cemiplimab in early-stage cSCC has demonstrated promising preliminary results and has prompted a phase III (NCT06585410) trial assessing the non-inferiority of intralesional cemiplimab compared to surgery in early-stage cSCC. Both studies are active with results pending [172,173,174].

Oncolytic viruses are genetically engineered viruses with anti-tumor activity and have shown promising results in patients with unresectable melanoma [175,176]. Several studies are underway investigating HSV-1 oncolytic viruses talimogene laherparepvec (T-VEC) and RP1 in solid organ transplant patients with advanced NMSCs. Early studies report modest response rates with favorable adverse effect profiles [177,178,179,180,181]. Oncolytic viruses are often injected intratumorally and may provide an effective option for IS patients who are intolerant to ICIs and other systemic therapies.

## 10. Conclusions

Immunosuppressed patients with cSCC represent a diverse group of patients who are at high risk for more aggressive disease and worse prognosis. Accordingly, care should emphasize prevention and early detection and involve a multidisciplinary team approach. Current staging and prognostic systems incompletely capture disease severity in IS patients, and decisions should be guided by individualized risk factors alongside these systems. Recent clinical trials have demonstrated the difficulty in comprehensively studying this patient population but have also shown promising new therapeutic modalities that may expand treatment options for IS patients with high-risk cSCC. 

## Figures and Tables

**Table 1:** Comparison of the two most commonly used staging systems for cutaneous squamous cell carcinoma. BWH, Bigham and Women’s Hospital. AJCC, American Joint Committee on Cancer.

T Staging	BWH	AJCC 8th Edition
T1	No high-risk factors	Tumor < 2 cm in greatest diameter
T2/T2a	1 high-risk factor	Tumor ≥ 2 cm but <4 cm in greatest diameter
T2b	2–3 high-risk factors	-
T3	≥4 high-risk factors or bone invasion	Tumor ≥ 4 cm in greatest diameter or minor bone invasion, or PNI, or deep invasion *
T4	-	a. Tumor with gross cortical bone/marrow invasion b. Tumor with skull bone invasion or skull-base foramen involvement
High-risk Factors	Tumor diameter ≥ 2 cm; PNI ≥ 0.1 mm nerve caliber; Poorly differentiated; Tumor invasion beyond subcutaneous fat	-
Comments	Does not include immunosuppression in the staging of cSCC. It is based on tumor-specific features only	Acknowledges immunosuppression in the subtexts as an adverse factor but does not include it in the TNM staging

Abbreviations: cSCC, cutaneous squamous cell carcinoma; PNI, perineural invasion; TNM, tumor, nodes, metastasis; * Deep invasion is depth > 6 mm or beyond subcutaneous fat.

**Table 2:** FDA-approved immunotherapy agents for cutaneous squamous cell carcinoma.

Drug (Brand)	Class	FDA Indication	FDA Approval	Clinical Trial	Key Findings
Cemiplimab (Libtayo)	anti-PD-1	Metastatic or locally advanced cSCC; not amenable to curative surgery or radiation	28 September 2018	EMPOWER-CSCC-1 (NCT02760498); Phase II	ORR 47% with a durable response ≥ 12 months
Pembrolizumab (Keytruda)	anti-PD-1	Metastatic/recurrent or locally advanced cSCC; not curable by surgery or radiation	24 June 2020 6 July 2021 *	KEYNOTE-629 (NCT03284424); Phase II	ORR 35–50% with a durable response ≥ 12 months
Cosibelimab (Unloxcyt)	anti-PD-L1	Metastatic or locally advanced cSCC; not candidates for curative surgery or radiation	13 December 2024	CK-301-101 (NCT03212404); Phase I	ORR 47–48% with a mean duration of response of 17.7 months in locally advanced disease

* Date of expanded indication for locally advanced cSCC.

## Data Availability

Not applicable.
